# Plate Waste Generated by Spanish Households and Out-of-Home Consumption: Results from the ANIBES Study

**DOI:** 10.3390/nu12061641

**Published:** 2020-06-02

**Authors:** Teresa Partearroyo, Mª de Lourdes Samaniego-Vaesken, Emma Ruiz, Javier Aranceta-Bartrina, Ángel Gil, Marcela González-Gross, Rosa M. Ortega, Lluis Serra-Majem, Gregorio Varela-Moreiras

**Affiliations:** 1 Departamento de Ciencias Farmacéuticas y de la Salud, Facultad de Farmacia, Universidad San Pablo-CEU, CEU Universities, Urbanización Montepríncipe, 28925 Alcorcón, Madrid, Spain; t.partearroyo@ceu.es (T.P.); l.samaniego@ceu.es (M.d.L.S.-V.); 2 CIBERESP (Consortium for Biomedical Research in Epidemiology and Public Health), 28029 Madrid, Spain; e.ruiz@externos.isciii.es; 3 National Center for Epidemiology, Carlos III Institute of Health, Avda. Monforte de Lemos, 5, 28029 Madrid, Spain; 4 Spanish Nutrition Foundation (FEN), 28010 Madrid, Spain; 5 Department of Food Sciences and Physiology, University of Navarra, Pamplona, 31009 Navarra, Spain; javieraranceta@gmail.com; 6 Department of Physiology, Faculty of Medicine, University of the Basque Country (UPV/EHU), 48940 Leioa, Vizcaya, Spain; 7 CIBEROBN, Biomedical Research Networking Center for Physiopathology of Obesity and Nutrition, Carlos III Health Institute, 28029 Madrid, Spain; agil@ugr.es (Á.G.); marcela.gonzalez.gross@upm.es (M.G.-G.); lluis.serra@ulpgc.es (L.S.-M.); 8 Department of Biochemistry and Molecular Biology II and Institute of Nutrition and Food Sciences, University of Granada, 18010 Granada, Spain; 9 ImFINE Research Group, Department of Health and Human Performance, Universidad Politécnica de Madrid, 28040 Madrid, Spain; 10 Department of Nutrition and Food Science, Faculty of Pharmacy, Madrid Complutense University, 28040 Madrid, Spain; rortega@ucm.es; 11 Research Institute of Biomedical and Health Sciences, University of Las Palmas de Gran Canaria, 35016 Las Palmas, Spain; 12 Service of Preventive Medicine, Complejo Hospitalario Universitario Insular Materno Infantil (CHUIMI), Canary Health Service, Las Palmas de Gran Canaria, 35016 Las Palmas, Spain

**Keywords:** plate waste, leftovers, food losses, catering services, household consumption, ANIBES Study

## Abstract

Food waste is a major environmental issue that must be tackled in order to achieve a sustainable food supply chain. Currently, in Spain there are no studies that examine the amounts and sources of plate waste (PW) produced by both household and out-of-home consumption. The present study aims to provide this information from a representative sample from the Spanish population. A total of 2009 individuals aged 9–75 years, from the ANIBES study (“anthropometric data, macronutrients and micronutrients intake, practice of physical activity, socioeconomic data and lifestyles in Spain”), completed a three-day dietary record, collected by a tablet device. Photographs of all foods and beverages consumed both at home and outside were taken before and after meals. Median PW across the total population was 7.3 (0.0–37.3) g/day and was significantly higher in females than males (*p* < 0.05) and in children vs. adolescents, adults, and elderly (*p* < 0.01). Regarding meals, PW across all age groups was higher at lunch (40%), dinner (27%), and breakfast (11%). The highest PW was observed for bread (25%) main courses (16%), first and second courses (15%), vegetables and fruits (12%), ready-to-eat meals (10%), cereals and grains (10%), oils and fats (10%), pulses (10%), meat products (8%), sauces and condiments (8%), and starters (8%). Our results reinforce the need for new strategies to focus on reducing plate leftovers, which are crucial from a nutritional, economic, and environmental point of view. Additionally, this evidence is important for relying on more accurate information on actual intakes when using dietary surveys.

## 1. Introduction

Back in 1987, the United Nations (UN) defined sustainability or sustainable development as “the one that meets the needs of the present without compromising the needs of future generations” [1]. At present, food systems are showing a major environmental impact worldwide, accounting for 19–29% of overall greenhouse gas emissions [2], and therefore compromising the sustainability concept and principles. Specifically, food waste (FW) is a major problem that must be tackled and minimized in order to achieve a more sustainable food supply chain [3]. According to the Food and Agriculture Organization (FAO), “food waste” can be defined as food that, although suitable for human consumption, is discarded as a result of negligence or the deliberate choice to throw it away [4]. The later a food product is lost or wasted in the supply chain, the higher the environmental cost, as more inputs are invested (processing, transport, cooking, etc.), and greenhouse gas emissions build up as the product moves through the supply chain [5]. Therefore, preventing the avoidable generation of FW in the supply chain represents the most beneficial approach to avoid food wastage [6]. It is difficult to know precisely how many foods that are suitable for consumption end up being discarded. Even though data on FW from European member states (EU) still have a high uncertainty level, the Fusions Project estimated that by 2012 an annual FW of 88 million tons in EU-28 member states would be produced, of which about 53% occur at household level and 12% at the food service or catering sector [7]. According to the 2014 FAO estimates, 1.3 billion tons of food was wasted annually (1/3 of the world’s production) [8].

The main source of FW in catering or food services (restaurants, bars, etc.) and institutions is the food that is not consumed, left on the plate, or leftovers [9,10]. There is a consensus in the literature on the definition of plate waste (PW) that is expressed as the quantity of an edible portion of served food that is discarded [11] or the fraction of FW represented by leftovers [12]. Specifically, in Sweden, Engström et al. [9] observed that PW was the major source of food loss, with about 11% to 13% of the amount of food served in food service institutions being thrown away.

Elimelech et al. [13] quantified a household food waste that accounted for 45% of total waste (573 g/day per capita), and importantly, they stated that 54% was identified as avoidable. There are many causes that can lead consumers to leave food on the plate [14]. For instance, in the catering sector, it was observed that up to 59% of clients could not eat all of what they were served, as serving sizes and portions were very large; and in this regard, 23% of plate leftovers (PL) were side dishes that consumers were unaware of when choosing their menus [15]. Furthermore, a number of studies have shown that reducing portion sizes is an effective measure toward decreasing FW. In a study by Freedman and Brochado [16], the authors indicated that decreasing the serving size of French fries by 50% resulted in a 31% reduction in PW per consumer. Moreover, Vermote et al. [17] showed that a downsizing of 20.9% in French fries portion resulted in a total intake reduction of 9.1% and a relative decrease of 66.4% of PW. This study did not only change portion size, but also the material and shape of the serving ware, as serving material has been previously shown to affect the quantity of PW [18]. In a study conducted by Kallbekken and Sælen [19] in hotel restaurants, they found that very simple measures, such as reducing plate size and informing guests they were allowed to help themselves more than once, reduced the amount of FW by 20%.

The latest report conducted in Spain by the Ministry of Agriculture, Fisheries and Food (MAPA) estimated a waste production, between January and December 2018, of 1339 million kg of food and drinks, which translates into and average FW production of 24.7 kg per capita/year. These data indicate that FW in Spanish households has increased by 8.9%, compared to data from 2017, a curious fact considering that most households state that they avoid throwing away food products [20] and in spite of the past and current economic crisis. At the household level, the latest data from the Panel of FW Quantification from Spanish Households developed by MAPA [20] showed that Spanish families discarded about 4.6% of purchased foods, a similar quantity of that reported by the “Food and society decision in 21st century Spain” study in adult population by CEU San Pablo University [21]. The latter indicates that, in relation to the measures undertaken by consumers to reduce FW, 85% of participants declared trying to reduce plate leftovers by planning their menus in advance, 84% planning their grocery shopping, and only 66% declared buying less amounts of food [21]. These data do not seem to confirm the ones from the Ministry report, in which 8 out of every 10 homes acknowledge discarding food and beverages. Namely, 81.5% of consumers discarded food products just as they were acquired, without any further processing or elaboration [20]; the main reasons for disposal were spoilage due to mold presence (29%), products past their “best before” or “use by” date (19%), leftovers from dining (14%), and preparation of food dishes above their actual needs (13%) [22]. The typical Spanish meal structure has changed over the last few years, and many traditions have been lost as dietary habits drift away from the Mediterranean Diet [23]. A regular daily menu mainly consists of a first course (legumes, vegetables, salads, soups, etc.), followed by a second course (fish, meat, pasta, etc.), a piece of bread, beverage, dessert, and coffee or infusion. Alternatively, main courses accompanied by side dishes (salads, vegetables, potatoes, etc.) can be consumed.

It should be underlined that, so far, most studies conducted in the Spanish population have focused on the overall waste generated by the Spanish consumers. However, to date, there are no studies that quantify and describe the food groups left by Spaniards per se on the plate. Hence, the present study aims to provide the information about the amount of household and out-of-home PW, accounting for relevant socioeconomic factors (habitat size, educational level, and family income), eating occasions, and different types of dishes consumed amongst different age groups of participants from the ANIBES study (“anthropometric data, macronutrients and micronutrients intake, practice of physical activity, socioeconomic data and lifestyles in Spain”) as a representative sample of the Spanish population.

## 2. Materials and Methods

Design, protocol, and methodology of the ANIBES study have been previously published [24], and full references can be found at the repository from the Spanish Nutrition Foundation (FEN) (http://www.fen.org.es/anibes/es/biblioteca). The design of the ANIBES study was cross-sectional, and stratified multistage sampling was used. Fieldwork was achieved at 128 sampling points across Spanish territory, aiming to include a sample size representative of all people living in Spain aged 9–75 years and living in municipalities of at least 2000 residents. The initial potential sample included 2634 individuals, and the final comprised 2009 (1013 men, 50.4%; 996 women, 49.6%). The youngest age groups (9–12, 13–17, and 18–24 years) included a “boost sample” to deliver *n* = 200 per age group (error ± 6.9%). Random plus boost sample involved 2285 subjects in total. Sample quotas were age groups (9–12, 13–17, 18–64, and 65–75 years), gender (male/female), and locality or habitat size (rural population: 2000 to 30,000 inhabitants; semi-urban population: 30,000 to 200,000 inhabitants; and urban population: >200,000 inhabitants). Additional factors considered for sample adjustment were education and monthly family income (0–1000 €, 1001–2000 €, and > 2000 €). The statistical description of the ANIBES population sample is included in Table 1.

**Figure 1:**
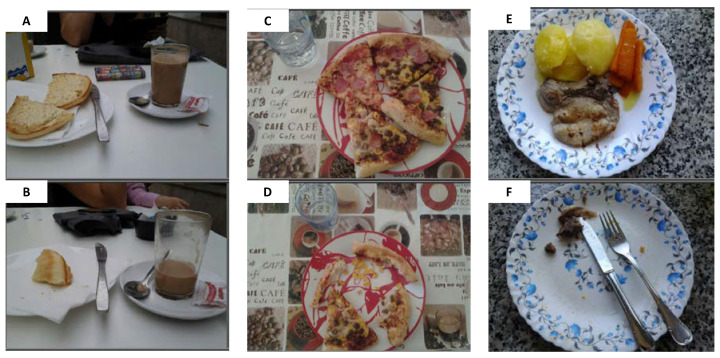
Selected examples of pictures taken by ANIBES study participants (before (**A**,**C**,**E**) and after consumption (**B**,**D**,**F**)).

Two pilot studies were completed before the main research period (mid-September 2013 to mid-November 2013), and participants were involved for two working-week days and one weekend day. The final protocol was accepted by the Ethical Committee for Clinical Research of the Region of Madrid, Spain. Subjects were provided a tablet (Samsung Galaxy Tab 27.0, Samsung Electronics; Suwon, Gyeonggi-do, South Korea), to facilitate recording all foods and drinks consumed over the course of those three days, by taking photographs of their meals, in their household and outside. It is described that self-report of dietary intake could be biased by the social desirability or social approval given to diet, and this might affect estimates in epidemiological studies [25]. In order to avoid self-report biases, no specific instructions were given to subjects regarding food consumption, so that they would eat as customarily as possible. Instructions given to participants can be found in Appendix A. Pictures of the dishes or food products were freely taken by participants, before and after each eating occasion, and these were used for performing comparisons within each participant’s plate. A fit-for-purpose software was developed to collect information from the tablets every two seconds, and comprised a database that was updated every 30 min. Food consumption was assessed with the use of the photographs (Figure 1), descriptions, and information collected, by a team of 14 dieticians/nutritionists, who codified foods and beverages and assigned portion weights (g). Our method is considered to be sound but still awaits proper validation.

Statistical analysis was performed as a descriptive analysis of the sample, with the main quantitative variables expressed through centralization and dispersion parameters. Results are reported as median (interquartile range) per group or as percentage. Variables were tested for normality, using a Kolmogorov–Smirnov test. Non-parametric data were analyzed by the Mann–Whitney U test, Wilcoxon test or the Kruskal–Wallis test, and when it resulted in differences, multiple comparisons between medians were studied by the Dunn test, to adjust for multiple comparisons and adjusted *p*-value with Bonferroni correction. Differences were considered significant at *p* < 0.05. Data analysis was performed with the SPSS 24.0 software package (IBM Corp., Armonk, NY, USA).

## 3. Results

Both household and out-of-home PW from total population and different gender and age groups are shown in Table 2 (and Supplementary Materials Figure S1). Female participants showed a significantly higher PW (11.7 (0.0–46.2) g/day) than males (4.0 (0.0–31.4) g/day) when the total population was analyzed (*p* < 0.05). However, by dividing population by age, we only observed that PW was significantly higher in women during adolescence and adulthood when compared to men. Moreover, household and out-of-home PW was highest among children aged 9 to 12 years (20.3 (4.3–59.7) g/day), followed by adolescents aged 13 to 17 years (12.7 (0.0–48.7) g/day), adults aged 18–64 years (8.3 (0.0–38.0) g/day), and elders aged 65–75 years (0.0 (0.0–13.3) g/day). Similarly, significant differences (*p* < 0.01) were observed amongst children, adolescent, adults, and the elderly. Furthermore, 19% of children, 28% of adolescents, 35% of adults, and 64% of elderly did not leave any PW (0 g/day) during the three-day record.

Table 3 shows the grams per day of PW that a person leaves a day on the plate, considering if food consumption is either at home or outside. Leftover quantities are greater when eaten at home than when eaten away from home. In addition, if we segment the places where people eat outside home, it can be seen that there is a slight tendency to leave a little more food on the plate when eating in institutions (schools, universities, jobs, etc.) than when eating at food service emplacements (restaurants, bars, etc.). It should be noted that out-of-home observations (*n* = 1407) do not add up when segmented into “Institutions” (*n* = 732) and “Food services” (*n* = 1144), because one participant might have lunch in an institution and then dinner at a restaurant (food service) on the same day, therefore increasing the number of observations.

**Figure 2:**
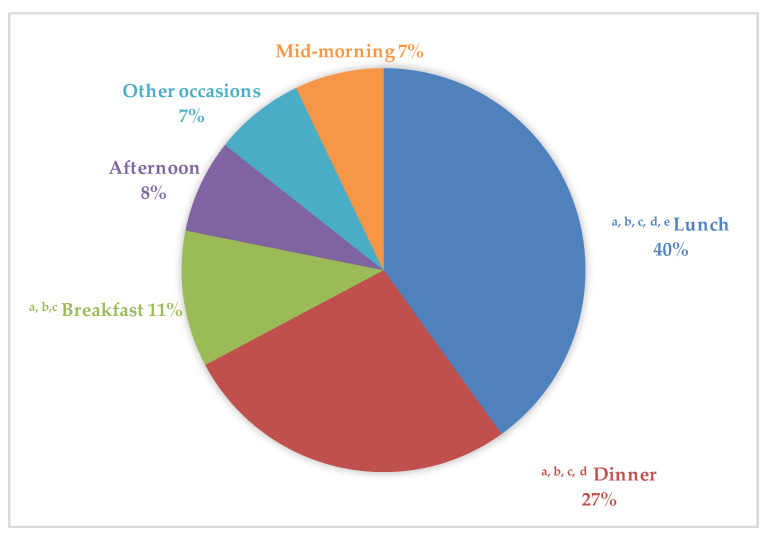
Percentage of household and out-of-home plate waste at different eating occasions, across the ANIBES study population. ^a^ *p* ≤ 0.001 mid-morning vs. breakfast, dinner, and lunch (Kruskal–Wallis and Dunn Test to adjust for multiple comparison). ^b^ *p* ≤ 0.001 other occasions vs. breakfast, dinner, and lunch (Kruskal–Wallis and Dunn Test to adjust for multiple comparison). ^c^ *p* ≤ 0.001 afternoon vs. breakfast, dinner, and lunch (Kruskal–Wallis and Dunn Test to adjust for multiple comparison). ^d^ *p* ≤ 0.001 breakfast vs. dinner and lunch (Kruskal–Wallis and Dunn Test to adjust for multiple comparison).

Figure 2 shows the distribution of food discarded over different eating occasions and the main meals that contributed to household and out-of-home PW across all groups were lunch (40%), dinner (27%), breakfast (11%), and afternoon (8%), with mid-morning and other occasions accounting for 7% each. Significant differences (*p* ≤ 0.001) were found between the central meals (breakfast, lunch, and dinner) and snacks (mid-morning, snack, and other occasions). However, the amount of waste generated between collations was very similar.

In Table 4, we can observe that there is more PW over working days than on weekends (*p* ≤ 0.001). In addition, average household and out-of-home PW over lunch was significantly higher (*p* ≤ 0.001) in children (9.3 (0.0–31.0) g/day) than in adults (0.3 (0.0–16.7) g/day) and elderly (0.0 (0.0–3.3) g/day), while adolescent females (8.7 (0.0–43.7) g/day) and adult females (2.0 (0.0–18.4) g/day) had a significantly higher PW than adolescent males (1.0 (0.0–17.7) g/day) and adult males (0.0 (0.0–13.3), (*p* ≤ 0.05 and *p* ≤ 0.01, respectively) (Table 5).

**Figure 3:**
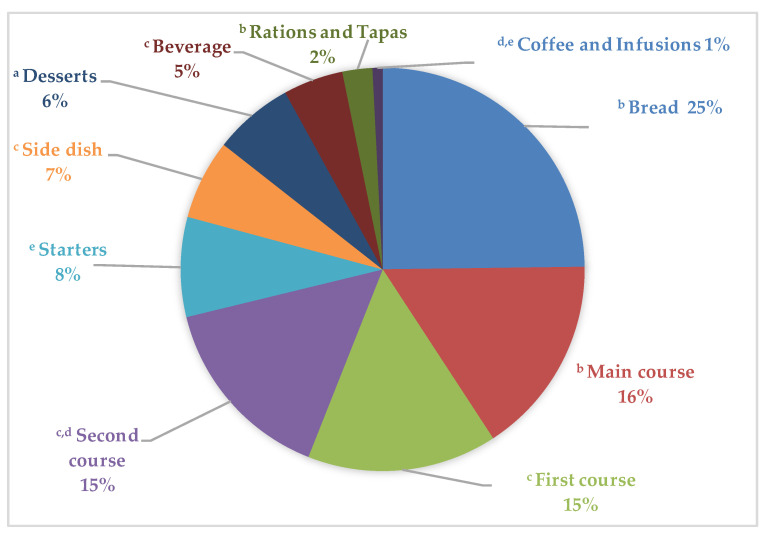
Distribution of plate waste across different menu components consumed by the ANIBES study population. Different superscript letters indicate statistically significant differences among groups (*p* < 0.05, Student–Newman–Keuls test).

Figure 3 displays the total PW generated by the ANIBES participants, accounting for the different menu components consumed throughout the day. The highest PW amounts corresponded to bread and similar products (25%), followed by main courses (16%), first and second courses (each 15%), and starters (8%), which were among the main contributors.

**Figure 4:**
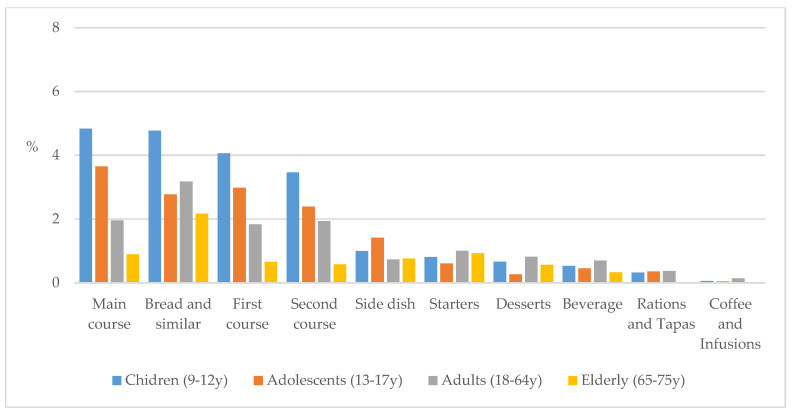
Percentage of plate waste across different menu components consumed by age groups from the ANIBES study population.

In addition, the largest amounts of leftovers attributable to children (4.8%) and adolescents (3.7%) were main courses, while adults and elders produced greater PW as bread and similar products, with 3.2% and 2.2%, respectively (Figure 4).

**Figure 5:**
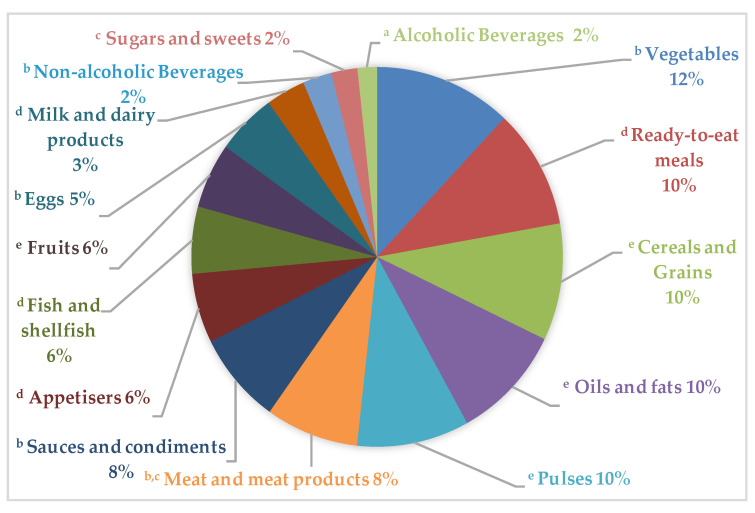
Distribution of food and beverage groups contributing to plate waste generated by the ANIBES study population. Different superscript letters indicate statistically significant differences among groups (*p* < 0.05, Student–Newman–Keuls test). Vegetables represented the major food group wasted amongst adolescents (5.2%) and adults (2.6%) (Figure 6), while children discarded a higher proportion of pulses or legumes (5.6%) and the elderly left meat and meat products (1.1%). Furthermore, household and out-of-home PW was highest amongst children across all food groups, except for fish and shellfish, which was higher in adolescents, followed by children and adults. Finally, it can be observed that the elderly was the age group who generated the lowest PW across all food categories.

The main food groups that contributed to PW across all age and gender groups were vegetables (12%), ready-to-eat meals (10%), cereals and grains (10%), oils and fats (10%), and pulses (10%), followed by meat and meat products (8%) and sauces and condiments (8%), as shown in Figure 5. On the other hand, groups such as fruits (6%), appetizers (salted snacks, olives, and pickles) (6%), eggs (5%), sugars and sweets, and alcoholic and non-alcoholic beverages (2% each) are less frequently discarded.

Table 6 illustrates household and out-of-home PW studied by socioeconomic factors. There were significant differences by habitat size, as higher leftover amount was observed in urban or rural zones when compared to semi-urban zones (*p* ≤ 0.01). Likewise, educational level (Table 6) did seem to influence population’s PW, as there was a significant difference between lower educational level compared to superior educational levels (*p* ≤ 0.01). Similarly, we did find a statistical difference in PW between lower (≤1000€ per month) and higher (≥2000€ per month) population income levels (Table 6).

## 4. Discussion

The present examination of nationally representative data delivers an insight into the meal plate leftovers or PW from Spanish households and out-of-home consumption by age group, gender, eating occasions, type of consumed dishes, habitat size, and educational and income level from the ANIBES Spanish population. We also identified the major food group sources that contributed to leftovers amongst the Spanish population.

### 4.1. Plate Waste by Total Population, Gender, and Age

The average level of PW identified amongst the ANIBES study participants was 7.3 g/d (1.73%). Similar results were published by Roe et al. [26], who observed that 3.3% of selected foods were left as PW among free-living individuals from the United States. However, other authors, such as Wansink and Van Ittersum [27], estimated a PW between 8% and 14% among customers at an all-you-can-eat Chinese buffet, and Freedman and Brochado [16] found a 18% PW from French fries in an all-you-can-eat university dining service. Other PW studies performed at university environments [28,29,30] yielded estimates of the volume of waste in the range of 63–124 g per meal. These values are much higher than our results, which may be explained in part by the different PW assessment methodologies applied, as most of these studies focused only on a single meal, usually lunch, while our study data were acquired and recorded overall, daily. In this regard, previous research found that higher household PW was generated at evenings rather than during midday or morning meals [31]. Concerning other key methodological issues, the present study is based on self-reported photographs, which may prove to be an improvement to written food diaries in which individuals could forget food items or wrongly describe ingredients or estimate quantities. Roe et al. [26] used a similar approach to ours to estimate PW from adults in free-living conditions by photographing consecutive meals with smartphones, but they also used laboratory-based meals with fixed food items and quantities and found PW in these conditions was significantly higher for same participants, showing a potential effect. However, there are drawbacks to self-reports to be considered, such as behavioral changes toward food consumption and choice over the period of study [32]. Our results also indicate that leftover amounts were significantly higher amongst women when compared to men. Nonetheless, we only found significant differences by gender during adolescence and adulthood. These results are in line with findings that indicate that women regularly leave more PW in laboratory meals where portion sizes are predetermined by researchers and similar for all respondents [26,33].

### 4.2. Plate Waste Generated at Different Eating Occasions by Age Groups

Concerning PW patterns, we found that lunch was the highest contributor amongst eating occasions, accounting for 40% of total PW discarded daily, while dinner accounted for 27%, and breakfast for only 11% of total PW. There are a number of possible explanations for the significant difference observed between lunch and the rest of eating occasions: Individuals do not invest enough time in having lunch due to busy schedules, work overload, etc.; serving sizes at catering services are sometimes excessive [16,17]; and, in the case of younger subjects, food selection and preference of specific groups could be responsible for a higher PW proportion. In addition, we found significant differences between the main meals and snack occasions. Conversely, other studies found that greater proportions of household PW were generated in the evening rather than over midday or morning meals [31]. In addition, our examination of PW across week or weekend consumption showed significantly higher PW during working days. Although PW variations between weekdays and weekends remain mostly unknown, former studies investigating weekly variation in dietary intakes have generally found less-healthy eating behavior during the weekend. Namely, larger intakes of total energy, fats, added sugars, different discretionary foods, and alcohol have been consistently observed over the weekend, in studies assessing dietary habits from Western populations [34,35,36,37,38,39,40,41]. It should also be acknowledged that, during working days, most people consume their main meal outside, in restaurants, or in their workplace, and that institutional kitchens might be more effective in calculating the right serving size of a dish, so a higher quantity of PW over these days might be related to potential time constraints of participants to finish their meals.

It is also important to acknowledge that PW might also lead to inadequate nutrient intakes, depending on the food group pattern that is discarded [31]. Notably, we found that the average household and out-of-home PW over lunch was significantly higher in children than in adults and the elderly. These results were expected, as children are a key group in which a high proportion tend to be very selective with what and how much they eat, presenting strong likes and dislikes of food, as well as not accepting new foods [42].

We also found that higher proportions of PW corresponded to bread, followed by main courses, and first and second courses; main courses were the largest PW attributable to children and adolescents, while bread was major amongst adults and the elderly. Bread is highly perishable and can dry quickly, becoming unappealing to consumers; also, the context of bread consumption (secondary to first and second courses) might lead to higher wastage. These data are in agreement with those obtained in the Quantification Panel of Food Waste in Spain [20], which indicated that leftovers from dishes such as soups, legumes, purees, creams, meat, and rice are those that end up most in the garbage, highlighting lentils, green salad, and Spanish potato omelet as those products with highest volume of food waste, and these are in turn typical foods consumed by the Spanish population as main, first, and second courses. Hence, one of the main objectives to consider in the design and enforcement of education programs to minimize waste is firstly to focus on preventing waste generation by a rational planification of food preparation that suits household or consumer needs (i.e., preparing less food) and secondly to impart how to reuse leftovers from already cooked food or to promote the composting of organic matter from the food waste of individuals, as well as catering companies, as a compensation measure. A recent review by Reynolds et al. [43] concluded that one of the most effective interventions to reduce food waste were those that changed type or size of plates (up to 57% food-waste reduction).

### 4.3. Dietary Food and Beverage Groups Contributing to Food Discarded by the ANIBES Study Population

There is a whole body of literature concerning food group categories that are most likely to be found in household garbage collections, with estimates of the percent of all purchases that end up discarded [20,44]. For instance, in England and Wales, the groups of foods (edible parts) and drinks with the highest waste rates included fresh vegetables and salads (28%), drinks (15%), and bakery products (11%) [44]. In Spain, fruits (31.9%), fresh vegetables (14.4%), dairy products (13.0%), and drinks (6.6%) are the most wasted products [20]. Nevertheless, in the current study, we found that vegetables (12%), ready-to-eat meals, cereals and grains, oils and fats, and pulses (10% each) were the main leftover constituents, while milk and dairy products and alcoholic and non-alcoholic beverages represented a much smaller proportion. These findings can be explained in part by the food consumption patterns we previously described from the ANIBES study population, where the vegetables group accounts for highest quantities (177.8 ± 112.9 g/day), only after milk and dairy products (257.2 ± 159.2) [45].

It is important to underline that we quantified the amounts of food left on the plate, not the proportion of food that was thrown away from what was purchased at households and out-of-home (i.e., waste that occurs after food preparation, refrigerator/freezer and cabinet cleanouts, discarding spoiled foods, etc.), as it was the case in previous studies in Spain. In our study, vegetables contributed to PW as the first food group among adolescents and adults, while in children, it was pulses, and in the elderly, it was meat and meat products. However, the contribution of PW reported in the present study is inconsistent with the data reported in earlier studies that examined PW amongst adolescents. For example, Roe et al. [26] found that the main sources that contributed to PW were sugar, sweets, and beverages, along with grain products in adults age 18 to 65 years from the United States. Likewise, Marlette et al. [46] found an average percentage waste of 44% for fruits, 24% for mixed dishes, 15% for milk, and 30% for vegetables among sixth-grade students. Smith et al. [47] conveyed similar results in middle schoolers (including 6th grade), in which they wasted an average of 43% of fruit (average of canned and fresh) and 31% of vegetables. On the one hand, it is significant to understand that these studies could have even more waste from vegetable dishes, since Reger et al. [48] reported that students consumed more vegetables at home than at school, where students felt the vegetables had poor taste and were prepared always the same way. On the other hand, it is again necessary to remember that these researchers only assessed single meals, frequently merely lunch.

### 4.4. Percentage of Household and Out-of-Home Plate Waste by Socioeconomic Factors from the ANIBES Study Population

Household sociodemographic characteristics could play a determinant role on food purchasing, as the purchase of vegetables and fruit may be particularly sensitive in terms of constraints over this food-group acquisition. Results from a study performed in Canada by Ricciuto et al. [49] emphasize these concerns, as researchers found that household size, composition, income, and education together explained 21%–29% of the variation in food purchasing related to lower-income households. In fact, the income and education gap that is observed amongst specific population groups, and the direct effect on food choice, must be considered in the design of public health interventions targeted at changing dietary behavior.

Demographic variables and their influence on food product availability and accessibility have already been examined in a number of studies worldwide [50], in Europe [51], and in our country [52,53,54], and there is consensus that, at present, these matters seem to have been overcome in most urban and semi-urban areas of Spain. In our study, we found that sociodemographic characteristics had a significant influence over food waste, as populations from lower-income and -education segments produced less PW than those of higher-income populations. Studies reporting high fruit and vegetable PW were carried out in low-income schools [48,55,56,57]. The differences found when compared to our study could be due to the fact that the former identified a number of factors, such as free or reduced-price lunch; dislike of taste, smell, or look of the food served; and the excessive amount of food served relative to age and gender, all of which could contribute to food waste [56].

### 4.5. Strengths and Limitations

Among the strengths of the present study, we can highlight its innovative character, since the methodology used to collect the presented data had the advantage of the use of mobile tablet technology to record food consumption and PW, therefore alleviating participant’s burden of identifying and/or weighing plate leftovers, as acknowledged in other studies [55,58,59]. In addition, and in contrast to our examination carried out throughout the day, former studies on PW have only examined a limited number of daily meals. Several potential limitations should be noted, however. Firstly, the participants’ burden of having to photograph their food throughout the day might have influenced their behavior. It is highly likely that the Hawthorne effect biased results. Foods that were selected, left on the plate, or thrown into the trash were not weighed, and quantities were estimated by using pictures, standard recall questionnaires, and food photograph atlas. Future studies could benefit from a combination of methods, to examine whether pictures analyses provide a good estimation of actual PW amounts. In fact, van Herpen et al. [60] showed that coding PW photographs was a valid method to measure household food waste, as comparisons of estimated weights with actual weights showed that coders could accurately estimate the weight of food waste from photographs, without general overestimation or underestimation, and with satisfactory correlations with actual weights. Nonetheless, there are more accurate digital photography methodologies, such as those that include the use of a reference card or image to standardize the angle of the photographs used to measure food intake/waste and therefore the recorded portion sizes [61]. This solution could accurately estimate the weight of food waste from photographs when participants included a fixed-size visual reference in each photo, which the current method was unable to use.

In addition, we did not assess if uneaten food or leftovers were stored by participants for later consumption or not; hence, our estimate of PW is an upper-bound estimate of the amount of food waste, as some could be consumed later. Finally, it is essential to recall that we estimated the amounts of food left on the plate, not the quantities thrown away from what was purchased for homes, such as the waste that is produced after food preparation, refrigerator/freezer and cabinet cleanouts, discarded spoiled foods, etc., as formerly remarked.

## 5. Conclusions

For the first time, our results quantify and describe the plate waste generated by a representative sample of the Spanish population and strengthen the importance of developing innovative strategies to decrease food waste through minimizing plate leftovers. Specifically, in our study, the most wasted foods included vegetables, followed by ready-to-eat meals, cereals and grains, oils and fats, and pulses. In addition, leftover quantities are greater when eaten at home than when eaten away from home. Therefore, we strongly believe that our results may contribute for a better understanding and awareness from the public health and sustainability concern. Finally, we wish to highlight that this represents a critical issue, not only from a nutritional, economic, and environmental point of view, but also for the requirement of relying on more accurate information on actual population intakes when assessing dietary surveys.

## Figures and Tables

**Figure 6:**
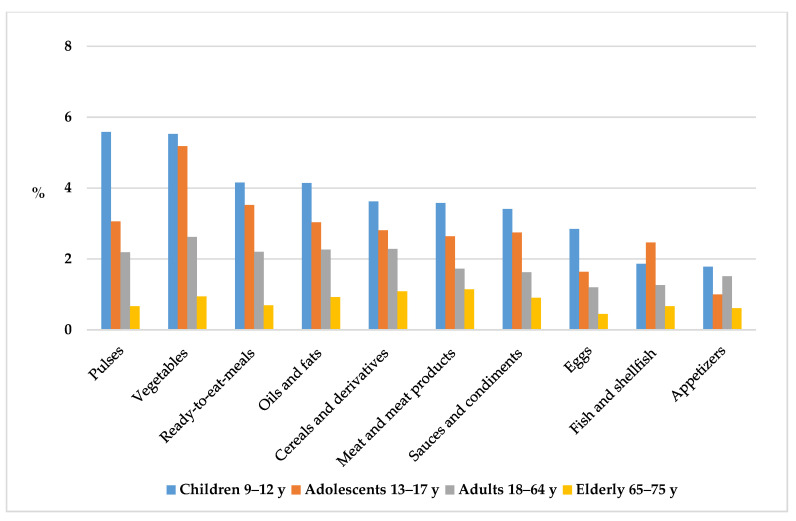
Food and beverage groups (%) contributing to plate waste across different age groups from the ANIBES study population.

**Table 1:** Statistical description of the ANIBES study population sample (“Anthropometric data, macronutrients and micronutrients intake, practice of physical activity, socioeconomic data, and lifestyles in Spain“).

		Total	%	Male	%	Female	%
**Age Group *n* = 2285**	Children (9–12 years)	213	9.3	126	10.9	87	7.7
	Adolescents (13–17 years)	211	9.2	137	11.8	74	6.6
	Adults (18–64 years)	1655	72.4	798	68.8	857	76.2
	Older adults (65–75 years)	206	9.0	99	8.5	107	9.5
**Level of education *n* = 2009**	Primary or less	744	37.0	379	37.4	365	36.7
	Secondary	859	42.8	435	42.9	424	42.6
	Tertiary or University	406	20.2	199	19.6	207	20.8
**Economical level *n* = 2009**	1000 € or less	405	20.2	196	19.4	209	21.0
	From 1001 to 2000 €	796	39.6	394	38.9	402	40.4
	Over 2000 €	320	15.9	163	16.1	157	15.8
	No answer	488	24.3	260	25.7	228	22.9
**Geographical distribution *n* = 2009**	Northeast	240	11.9	121	11.9	119	11.9
	Levante (East)	335	16.7	176	17.4	159	16.0
	South	443	22.1	218	21.5	225	22.6
	Central	191	9.5	107	10.6	84	8.4
	Norwest	152	7.6	77	7.6	75	7.5
	North central	162	8.1	80	7.9	82	8.2
	Canary Islands	93	4.6	44	4.3	49	4.9
	Madrid Metropolitan Area	264	13.1	133	13.1	131	13.2
	Barcelona Metropolitan Area	129	6.4	57	5.6	72	7.2

**Table 2:** Household and out-of-home plate waste generated by the ANIBES study population.

			*N*	Plate Waste (g/day)
**Total Population (9–75 years)**		Total	2009	7.3 (0.0–37.3)
		Men	1013	4.0 (0.0–31.4)
		Women	996	11.7 ** (0.0–46.2)
**Age**	9–12 years	Total	213	20.3 ^a^ (4.3–59.7)
		Men	126	20.3 (2.0–58.3)
		Women	87	23.3 (7.0–69.2)
	13–17 years	Total	211	12.7 ^b^ (0.0–48.7)
		Men	137	6.0 (0.0–40.4)
		Women	74	32.7 ** (1.0–73.8)
	18–64 years	Total	1655	8.3 ^c^ (0.0–38.0)
		Men	798	3.3 (0.0–30.0)
		Women	857	13.0 ** (0.0–47.3)
	65–75 years	Total	206	0.0 ^d^ (0.0–13.3)
		Men	99	0.0 (0.0–13.3)
		Women	107	0.0 (0.0–11.7)

Values are median (interquartile range) per group. Different superscript letters indicate statistically significant differences between ages, *p* ≤ 0.001 (Kruskal–Wallis test), and ** indicate statistically significant differences between gender (all differences are *p* ≤ 0.01; Mann–Whitney U test). ANIBES: Anthropometric data, macronutrients and micronutrients intake, practice of physical activity, socioeconomic data, and lifestyles in Spain.

**Table 3:** Plate waste (PW g/d) originated at different places from the ANIBES study population.

	Mean ± SD	Median (P_25_–P_75_)		Mean ± SD	Median (P_25_–P_75_)
Household *n* = 2006	23.9 ± 48.4	4.3 (0.0–30.0)	Household *n* = 2006	23.9 ± 48.4	4.3 (0.0–30.0)
Out-of-home *n* = 1407	17.2 ± 48.4	0.0 *** (0.0–16.7)	Institutions *n* = 732	6.0 ± 24.4	0.0 ^+++^ (0.0–0.0)
			Food services *n* = 1144	5.6 ± 22.5	0.0 ^+++^ (0.0–0.0)

Institutions include school, university, and workplace canteens. Food services include restaurants, fast-food, bars, etc. Results are expressed as the mean ± standard deviation (SD) and median (P25–P75) per group. *** *p* ≤ 0.001 vs. *household (Mann–Whitney test)*. ^+++^ *p* ≤ 0.001 vs. *household (Kruskal–Wallis chi-squared test)*.

**Table 4:** Household and out-of-home plate waste (PW. g/d) by week schedule amongst the ANIBES study population.

	Mean ± SD	Median (P_25_–P_75_)
Working days	28.7 ± 57.7	3.5 *** (0.0–33.0)
No working days	30.5 ± 95.1	0.0 (0.0–20.0)

Results are expressed as the mean ± standard deviation (SD) and median and P25–P75 (in brackets) per group. *** *p* ≤ 0.01 (Mann–Whitney test).

**Table 5:** Plate waste (PW, g/day)) originated at different eating occasions by age groups from the ANIBES study population.

KERRYPNX		Children (9–12 year)			Adolescents (13–17 year)			Adults (18–64 year)			Elderly (65–75 year)		
		Total	Women	Men	Total	Women	Men	Total	Women	Men	Total	Women	Men
		*n* = 213	*n* = 87	*n* = 126	*n* = 211	*n* = 74	*n* = 137	*n* = 1655	*n* = 857	*n* = 798	*n* = 206	*n* = 107	*n* = 99
**PW after dinner (g/day)**	Median	0.0	2.3	0.0	0.0	0.0	0.0	0.0	0.0	0.0	0.0	0.0	0.0
	(P25–P75)	(0.0–14.7)	(0.0–14.0)	(0.0–16.3)	(0.0–8.3)	(0.0–19.3)	(0.0–5.0)	(0.0–5.0)	(0.0–6.7)	(0.0–3.3)	(0.0–0.0)	(0.0–0.0)	(0.0–0.0)
	Mean ± SD	13.7 ± 26.7	12.7 ± 21.4	14.5 ± 30.0	11.2 ± 28.5	14.0 ± 26.6	9.7 ± 29.4	8.2 ± 21.9	8.6 ± 19.5	7.9 ± 24.3	4.0 ± 18.6	4.8 ± 24.2	3.2 ± 9.4
**PW after lunch (g/day)**	Median	9.3 ^a^	10.0	8.2	3.0 ^a.b^	8.7 *	1.0	0.3 ^b^	2.0 **	0.0	0.0 ^c^	0.0	0.0
	(P25–P75)	(0.0–31.0)	(0.0–39.2)	(0.0–28.8)	(0.0–23.6)	(0.0–43.7)	(0.0–17.7)	(0.0–16.7)	(0.0–18.4)	(0.0–13.3)	(0.0–3.3)	(0.0–0.0)	(0.0–10.6)
	Mean ± SD	21.8 ± 30.1	26.5 ± 35.5	18.6 ± 25.4	18.8 ±33.9	27.1 ± 43.4	14.3 ± 26.5	14.8 ± 37.0	15.7 ± 28.4	13.8 ± 44.5	7.8 ± 18.3	7.4 ± 19.9	8.2 ± 16.4
**PW after breakfast (g/day)**	Median	0.0	0.0	0.0	0.0	0.0	0.0	0.0	0.0	0.0	0.0	0.0	0.0
	(P25–P75)	(0.0–1.3)	(0.0–1.3)	(0.0–1.7)	(0.0–0.0)	(0.0–2.0)	(0.0–0.0)	(0.0–0.0)	(0.0–0.0)	(0.0–0.0)	(0.0–0.0)	(0.0–0.0)	(0.0–0.0)
	Mean ± SD	2.3 ± 5.6	2.0 ± 5.3	2.4 ± 5.8	3.1 ± 9.2	4.0 ± 9.0	2.6 ± 9.3	2.0 ± 9.2	3.0 ± 12.0	0.9 ± 4.5	0.5 ± 2.4	0.5 ± 2.8	0.5 ± 2.0
**PW after afternoon snack (g/day)**	Median	0.0	0.0	0.0	0.0	0.0	0.0	0.0	0.0	0.0	0.0	0.0	0.0
	(P25–P75)	(0.0–0.0)	(0.0–0.0)	(0.0–0.0)	(0.0–0.0)	(0.0–0.0)	(0.0–0.0)	(0.0–0.0)	(0.0–0.0)	(0.0–0.0)	(0.0–0.0)	(0.0–0.0)	(0.0–0.0)
	Mean ± SD	2.6 ± 9.4	2.3 ± 8.7	2.7 ± 9.9	0.9 ± 5.1	2.5 ± 8.4	0.1 ± 0.7	1.1 ± 14.4	1.6 ± 19.3	0.6 ± 5.2	0.5 ± 3.9	0.7 ± 5.4	0.1 ± 1.0
**PW after morning snack (g/day)**	Median	0.0	0.0	0.0	0.0	0.0	0.0	0.0	0.0	0.0	0.0	0.0	0.0
	(P25–P75)	(0.0–0.0)	(0.0–0.0)	(0.0–0.0)	(0.0–0.0)	(0.0–0.0)	(0.0–0.0)	(0.0–0.0)	(0.0–0.0)	(0.0–0.0)	(0.0–0.0)	(0.0–0.0)	(0.0–0.0)
	Mean ± SD	1.1 ± 6.6	1.7 ± 9.5	0.7 ± 3.4	0.6 ± 6.5	1.8 ± 10.8	0.0 ± 0.0	1.2 ± 10.5	1.3 ± 10.5	1.0 ± 10.5	0.7 ± 6.2	0.8 ± 8.1	0.6 ± 3.1
**PW after other moments (g/day)**	Median	0.0	0.0	0.0	0.0	0.0	0.0	0.0	0.0	0.0	0.0	0.0	0.0
	(P25–P75)	(0.0–0.0)	(0.0–0.0)	(0.0–0.0)	(0.0–0.0)	(0.0–0.0)	(0.0–0.0)	(0.0–0.0)	(0.0–0.0)	(0.0–0.0)	(0.0–0.0)	(0.0–0.0)	(0.0–0.0)
	Mean ± SD	0.7 ± 5.9	1.4 ± 9.2	0.2 ± 0.9	1.9 ± 12.0	2.4 ± 13.2	1.7 ± 11.4	2.6 ± 19.9	2.6 ± 15.5	2.5 ± 23.8	0.1 ± 0.8	0.2 ± 1.2	0.0 ± 0.0

Results are expressed as the mean ± standard deviation (SD), and median and P25–P75 (in brackets) per group. Different superscript letters indicate statistically significant differences between ages, *p* ≤ 0.001 (Kruskal–Wallis test), and * indicates statistically significant difference between gender, (all differences are *p* ≤ 0.05; Mann–Whitney U test). ANIBES: Anthropometric data, macronutrients and micronutrients intake, practice of physical activity, socioeconomic data, and lifestyles in Spain.

**Table 6:** Plate waste (PW, g/day) segmented by socioeconomic factors from the ANIBES study population.

		Mean ± SD	Median (P_25_–P_75_)
Habitat size	Rural (*n* = 682)	31.5 ± 69.9	7.5 (0.0–39.0
	Semi-urban (*n* = 683)	26.0 ± 43.7	5.0 ** (0.0–33.3)
	Urban (*n* = 644)	30.4 ± 49.0	10.5 (0.0–40.3)
Educational level	Primary or lower (*n* = 744)	25.6 ± 46.9	5.0 (0.0–32.6)
	Secondary (*n* = 859)	32.0 ± 65.4	9.0 ^++^ (0.0–39.0)
	University (*n* = 406)	30.2 ± 46.2	10.0 ^++^ (0.0–41.3)
Monthly family income	0–1000 € per month (*n* = 405)	26.8 ± 49.0	5.3 (0.0–31.0)
	1001–2000 € per month (*n* = 796)	31.3 ± 60.9	10.0 ^#^ (0.0–41.7)
	> 2000 € per month (*n* = 320)	31.1 ± 55.5	10.3 ^#^ (0.0–39.0)

Results are expressed as the mean ± standard deviation (SD), and median and P25–P75 (in brackets) per group. ** *p* ≤ 0.01 vs. urban (Kruskal–Wallis chi-squared test). ^++^ *p* ≤ 0.01 vs. primary or lower (Kruskal–Wallis chi-squared test). ^#^ *p* ≤ 0.05 vs. 0–1000 € per month (Kruskal–Wallis chi-squared test).

## Supplementary Materials

The following are available online at https://www.mdpi.com/2072-6643/12/6/1641/s1. Figure S1: Household and out-of-home plate waste generated by total, gender, and age group from the ANIBES study population (Y-axis is plotted in logarithmic scale).

## Appendix A

**INSTRUCTIONS FOR THE CORRECT REALIZATION OF THE PHOTOS BEFORE AND AFTER CONSUMING EACH PRODUCT**

The ANIBES Study participants received the following instructions after signing the informed consent for inclusion.

**It is very important that the photos you take are sharp. Please note the following considerations:**

1. If you are consuming prepared, precooked dishes, etc., it is very important that the entire package shows in the image and we are able to read the corresponding brand perfectly. Likewise, if it is beverage containers, water, soft drinks, bottled juice, etc., it is very important that we can read the entire container and read the corresponding brand perfectly.
2. In the case of consuming chewing gum and/or candy, food supplements (shakes, envelopes, syrups, pills, bars, etc.) or vitamin supplements, the brands and composition of the products must also be clear (with or without sugar, ingredients, etc.). If there are leftovers, you must take the final photo of the leftovers. If there are no leftovers, the final photo will show empty containers, wrappers, etc.
3. In case of repeating any dish, drink or any other type of product, you must also photograph it, both before starting to consume it and once consumed or when you are no longer going to continue eating from that dish or drinking that drink.

**Water and other drinks**

1. If it is tap water or natural fruit juice squeezed at that time, either prepared at home or outside it, the photo should show the glass, jug or bottle with the tap water and/or the juice natural squeezed before and after consuming it.
2. If you share a jug or container of tap water with another person, then you must photograph, before and after, each and every one of the glasses of tap water that you drink individually, and then in the final description of the intake of that moment, write down 1 or 2 or 3, etc., glasses of tap water.
3. If you are in a house and tap water is served directly into a glass, then you should photograph the glass before drinking it, and also afterward, to know if you drank it whole or left some liquid, in addition to writing down, in the final description question of intake, the number of glasses of tap water you have taken at that time.
4. It is very important that you photograph each of the glasses of tap water taken before and after drinking them, and also note the number of glasses of tap water consumed in the final question of the detailed intake description. We want to remind you because the glasses of tap water are easy to forget, both for photographing them and for pointing them out in the detailed description below.
5. Other possible water consumption. For example, if you are at work or studying at home or elsewhere and start taking a bottle of water, either from the tap or bottled, you should take a photo of the container before you start drinking and another at the end, when the container is finished or when you no longer want to drink more water, even if you have spent a long time drinking the bottled water from the container, bottle, jug, etc. The intake description question should include whether it is tap water or bottled and, if so, what brand.
6. In the consumption of mini tetra Brik of juices, soda cans or any other container where you cannot see the amount of liquid consumed, you must also take the photo of end of intake even if you do not see what is consumed and, later, in the description question end of intake should reflect whether the whole can was taken or left half. For example, if you ordered two cans and had one whole and did not finish the other, you will note that you consumed one and a half cans. That is, the scale will be whole or half can, without going into greater detail.
7. Coffees and teas. If you drink coffee or an infusion at the end of the meal, also take two photographs: one at the beginning and one at the end of the intake. Make sure that the size of the cup and, if possible, the brand of the infusion is clearly visible.
8. Other types of drinks consumed in glasses or cups. You must take a photo of the bottle from which the drink comes out, to see the brand, and then a photo before and after each and every one of the cups or glasses you take. In the final description of the intake, write down the number of cups or glasses that have been taken.
9. Combined drinks. If you drink combination drinks, you must first take a photo of each of the bottles of the liquids you are going to combine, where the marks of the bottles of the drinks you combine can be seen clearly.
10. Then you will take a photo at the beginning and another at the end of the glass in which the combined drink was taken, to make it clear what its size is and if you finished the drink or not.
11. Please repeat this process for each of the combined glasses that you consume. In the final intake description question, please write down the number of combined drinks you had.
