# Supplementary material for: Plate Waste Generated by Spanish Households and Out-of-Home Consumption: Results from the ANIBES Study
Source: Nutrients. 2020 Jun 2;12(6):1641. doi: 10.3390/nu12061641 (PMC7352750; doi:10.3390/nu12061641)
Supplement: Supplementary file 1 [file nutrients-12-01641-s001.pdf]

Supplementary material

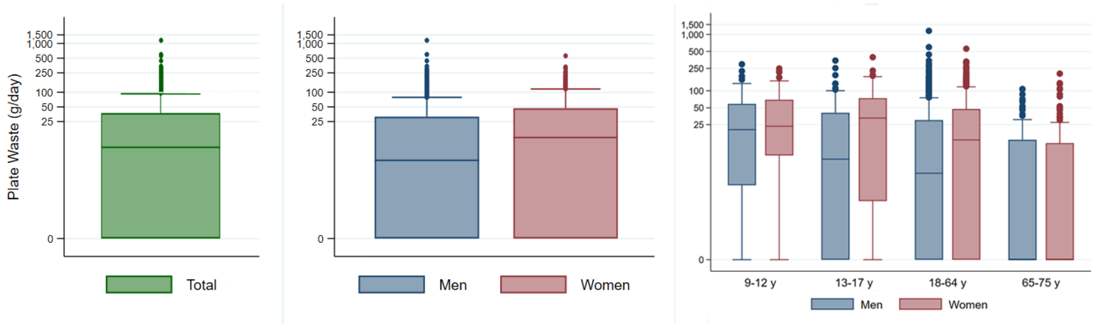

Figure S1. Household and out-of-home plate waste generated by total, gender and age group from the ANIBES study population (Y-axis is plotted in logarithmic scale).
